# Badland distribution as a marker of rapid tectonic activity

**DOI:** 10.1038/s41598-025-00795-9

**Published:** 2025-05-07

**Authors:** Ci-Jian Yang, Jens M. Turowski, Boris Gailleton, Chun-Wei Tseng, Chuan-Min Chao, Ray Y. Chuang

**Affiliations:** 1 Department of Geography, National Taiwan University, No. 1, Sec. 4, Roosevelt Rd, Taipei, 10617 Taiwan (R.O.C.); 2 German Research Centre for Geosciences (GFZ), Telegrafenberg, 14473 Potsdam, Germany; 3 Geosciences Rennes, University of Rennes 1, 35042 Rennes, France; 4 Taiwan Forestry Research Institute, No. 53, Nanhai Rd., Zhongzheng Dist, Taipei City, 10066 Taiwan (R.O.C.)

**Keywords:** Geomorphology, Natural hazards

## Abstract

Tectonically driven processes, such as uplift and horizontal advection of rock mass, are important controls on landscape form and dynamics. Available fluvial topographic indicators are intended to be applied to slowly evolving systems over millennial time scales, which may prevent extracting short–term rapid tectonic effects (~ 1ky). Fast–eroding badlands provide an opportunity to study landscape response to uplift over short time scales. Here, using 1 m airborne LiDAR topographic data in the tectonically active area of southwestern Taiwan, we find that badlands occur within the most rapidly uplifting areas of the landscape, where uplift rates exceed 7 mm y^− 1^. Furthermore, badlands are predominantly observed within the altitude range that coincides with rapid uplift at 1.3–1.5 kyBP, implying that they formed in response to meso–scale (< 100 km^2^) rapid incision of tributaries. We demonstrate how the existing landscape–archive framework, connecting dimensionless erosion rate and relief for hillslopes, can be used on highly erodible badland morphology. As such, badlands can provide archives of uplift history for transient landscapes, which allows to target shorter timescales than previously possible.

**Supplementary Information:**

The online version contains supplementary material available at 10.1038/s41598-025-00795-9.

## Introduction

Tectonic activity alters landscape form and dynamics and thus mountain building and erosion^1–3^. Moreover, it can lead to various natural disasters, thereby increasing the risk to the lives of those residing in affected areas^4^. Constraining spatio–temporal patterns of tectonic uplift help to reveal the mechanisms of regional landscape evolution and supports hazard mapping. Natural landscapes achieve a steady state by adjusting to tectonic uplift over a range of timescales. As such, topography records information on tectonic uplift. For example, fluvial geomorphic indices derived from natural landscapes allow us to obtain tectonic uplift rates and patterns over timescales of hundreds of thousands of years^5–9^. Yet, the most popular of these indices, channel steepness, does not apply to hillslopes and debris flow channels. Alternatively, the landscape archive framework exploiting hillslope morphology, has been demonstrated to reflect long–term landscape evolution^10–12^. With a shorter response time than the river system, this framework may be well suited to constrain the effect of tectonics on topography in low–relief areas with weak lithologies and high erosion rates.

Badlands are fast–evolving landscapes and are especially sensitive to environmental change^13–15^. Badlands typically form due to soft lithologies^16^ and climate oscillations^17^. Tectonic uplift changes the base level on a regional scale, resulting in incision that propagates upstream through river networks, thereby initiating the expansion of gully and badland systems^18^. At the same time, river terraces formed at the onset of the incision can provide temporal reference markers. Thus, once their origin and evolution are understood, fast–eroding landscapes such as badlands, can serve as suitable archives for reconstructing regional uplift histories over short timescales. Here, we suggest that the spatial distribution of badlands reflects the pattern of tectonic activity, and we demonstrate the applicability of the landscape–archive framework to a fast-evolving system.

**Fig. 1:**
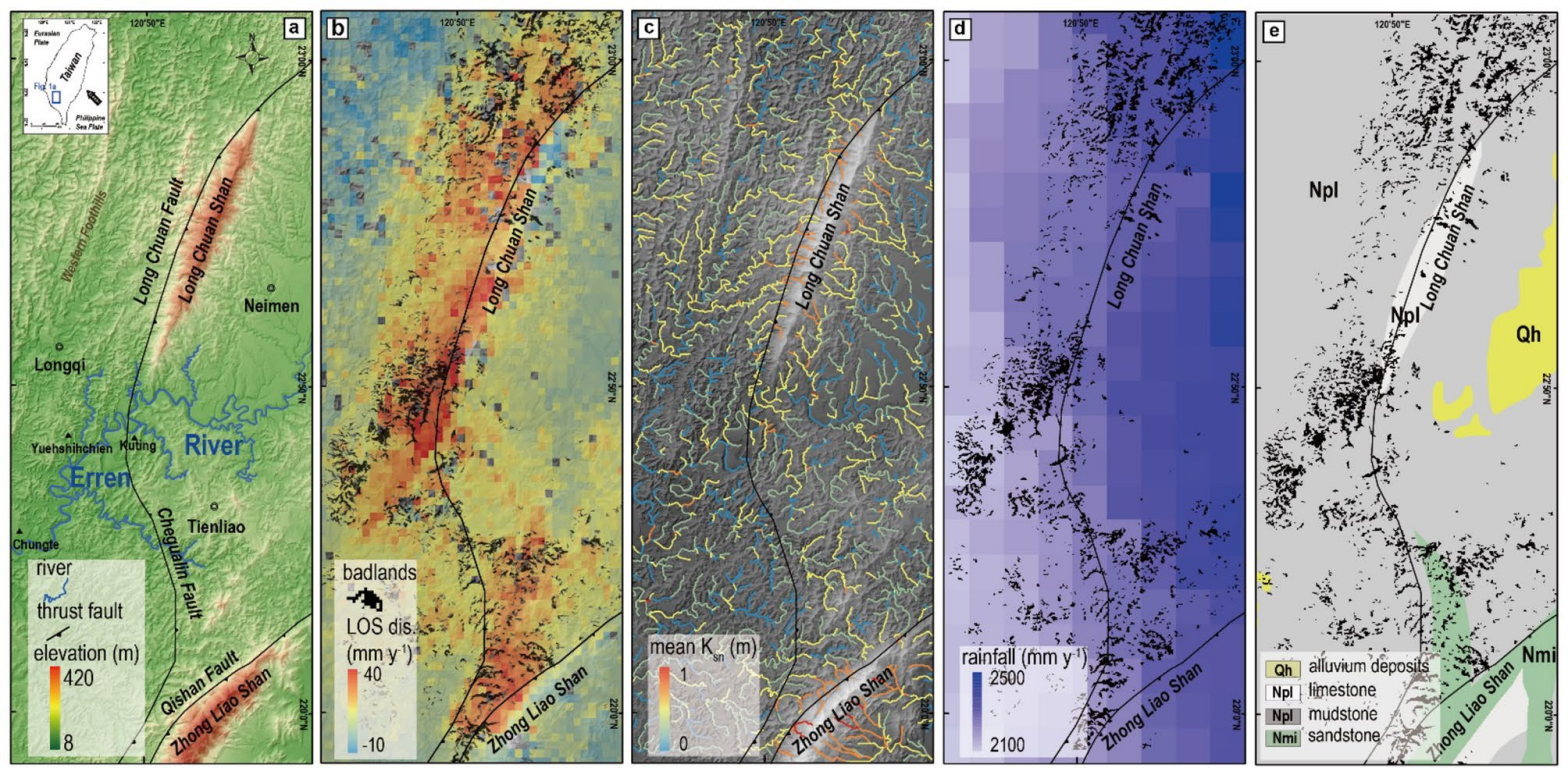
Study site and background information. (**a**) Color–shaded relief map showing the distribution of major faults. (**b**) InSAR results based on ALOS–1 data, showing the LOS displacement, which, shows spatial correlation with mapped badland area. (**c**) The normalized steepness index of channels (k_sn_, *θ* = 0.45) on a shaded relief map. (**d**) Grid–averaged annual rainfall map (2000–2010) (source: Central Weather Bureau, https://asrad.pccu.edu.tw/dbar/, last access: 5 May 2025). (**e**) Regional lithological map (source: Geological map of Taiwan scale 1:50,000, published by Geological Survey and Mining Management Agency, 2013, originally known as the Central Geological Survey, https://www.gsmma.gov.tw/nss/p/index, last access:  5 May 2025).

To elucidate the links between regional tectonic uplift and the distribution of badlands, we study a site located along the active Longchuan fault, on the frontal zone of the Taiwan orogenic belt (Fig. 1a). The Plio–Pleistocene Gutingkeng formation is the primary geological formation. Extending over several kilometers in depth, the main lithology consists of poorly consolidated mudstones, composed of clay (27.6%) and silt (70.6%)^19^. Differential erosion and fault activity have been identified as the mechanisms behind the formation of the Longchuan and Qishan faults^20^, and the associated two regional highlands. These are distinct from the badland landscape evolution caused by short-term tectonic uplift, and we will not discuss them in this study. The Longchuan fault is divided into two segments. The northern segment is a reverse fault in NNE–trending, while the southern segment of the Longchuan fault, also known as the Chegualin fault is characterized as a left–lateral fault with a reverse component^20^. The timing of tectonic uplift can be constrained since the middle Holocene when multiple river terraces developed in the Erren River, some of which are dated^21,22^. Channel incision rates into bedrock calculated from the terraces at Yuehshihchieh (Fig. 1a) were 7.6 mm y^− 1^ during ca. 5.7–2.5 ky before present (BP), 5 cm y^− 1^ during 1.5–1.3 kyBP, 2.6 cm y^− 1^ during 1.3–1.0 kyBP and 1.2 cm y^− 1^ since 1 kyBP^21^. The present surface uplift rate (Fig. 1b) has been measured at a maximum of 40 mm y^− 1^ using persistent scatterers interferometry with advanced land observing satellite synthetic aperture radar images from 2007 to 2010 (see details in methods). Neotectonics^21,22^, the development of a diapiric anticline^23,]^ and duplex structure combined with the high–pressure layers within the mudstone formation^24^ are thought to contribute to this uplift, but the detailed mechanism is still unknown (see details in supplementary). Current topography has been linked to long–term uplift beyond decadal time scales^21–23^. In addition, badlands in southwestern Taiwan have experienced a denudation rate of ~ 11 mm y^− 1^ at the millennial scale^25^ and hillslope erosion rates can reach 90–300 mm y^-1^^14,26^.

To connect hillslope morphology with tectonic activity, we used TopoToolbox^27^ to extract the necessary parameters for dimensional variables^28^ from 1 m topographic data acquired by airborne LiDAR (see details in methods). To convert altitude into absolute time, we assume that tectonic uplift is uniformly distributed across the spatial domain. In addition, we assume that the stream network rapidly responds to tectonic uplift due to lithological weakness, with a response timescale of decades. Then, all points in the landscape at the same elevation as a particular terrace have the same age as this terrace. Subsequently, we can use hillslope morphology to reconstruct the uplift rate along altitudes of badlands. We use a 1–D landscape evolution model for hillslope morphology to calculate the time-series erosion rate and to invert for uplift rate (see details in methods).

## Results and discussion

### Badlands are concentrated in certain vertical and horizontal areas

Based on a total of 133 million pixels, the average slope gradient of the study site is 22.57 ± 14.37^o^, and the badland area of 11.9 km^2^ accounts for 9% of the total area. In many landscapes, river bed slope (*S*) scales with drainage (*A*) according to a power law, following an equation of the form:

1 $$\:\text{S}={k}_{sn}{A}^{-\theta\:}$$

Here, *θ* is a dimensionless exponent known as the concavity index, while *k*_*sn*_ is known as the steepness index, which has been interpreted as a proxy for rock uplift rate in steady-state landscapes^6,7^. Although the highest decadal uplift rate is ca. 40 mm yr^− 1^ along the hanging wall of the fault, the steepness index *k*_*sn*_ is generally low and does not correlate with the uplift pattern (Fig. 1b) at the study site (R^2^ = 0.11, *p* < 0.05) (Fig. 1c). This indicates that the channels have not reached a steady state slope, possibly due to the influence of ongoing rapid incision into weak bedrock.

**Fig. 2:**
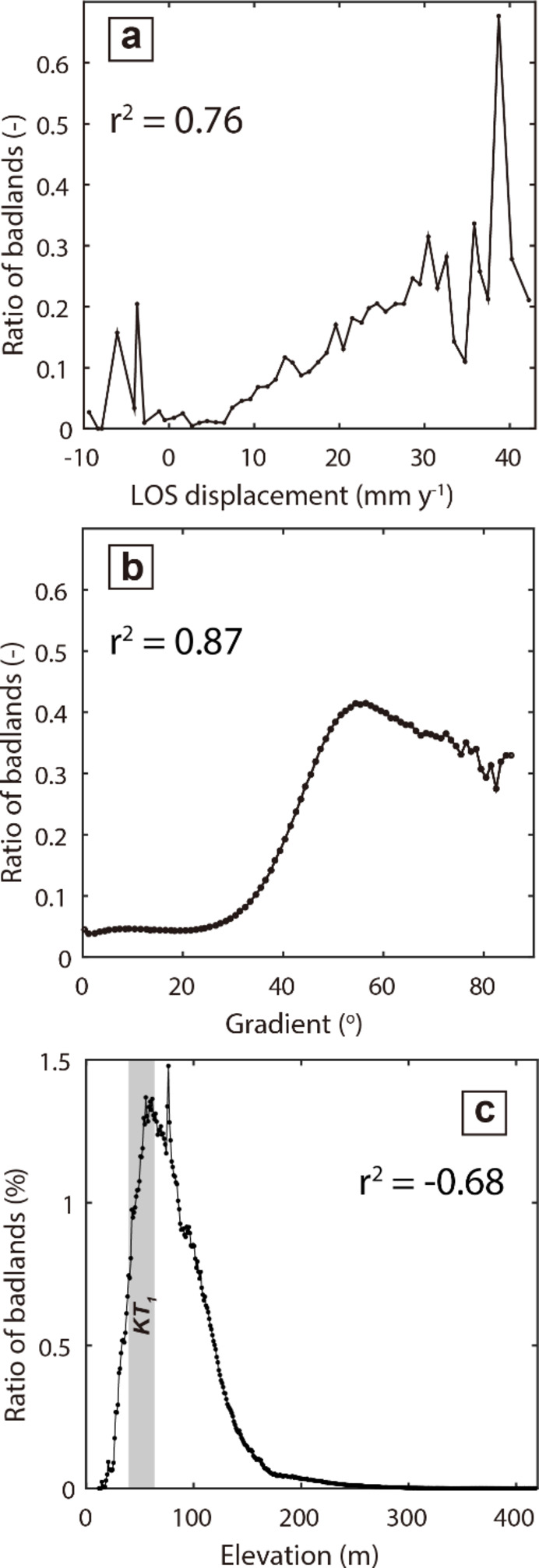
Correlation of hillslope gradient, LOS displacement, and the ratio of badlands area. (a) Correlation of LOS displacement and ratio of the badland area. (**b**) Correlation of gradient and ratio of the badland area. (**c**) Correlation of elevation (0–100 m) and the ratio of the badland area, KT_1_ denotes the position of a river terrace. The correlation coefficient is calculated at a significance level of 95%.

Instead, the ratio of badland area positively correlates with surface velocity for surface velocity above 7 mm yr^− 1^ and reaches a maximum value of ~ 0.68 at a surface velocity of 39 mm yr^− 1^ (Fig. 2a). For median slope gradients above 27^o^, the ratio of badland area is also positively correlated with the median gradient, and reaches a maximum value of 0.41 at gradients of around 54^o^ (Fig. 2b), and decreases for higher gradients. The ratio of badland area increases from 24 m of elevation to a peak ratio of 0.13 at 40 m and decreases to 0.09 for elevations exceeding 60 m (Fig. 2c).

Our dataset contains a total of 6316 hillslopes, with a mean slope gradient of 0.54 ± 0.22 in tangent value (28.43 ± 12.34^o^), and drainage areas ranging from 2000 to 33,245 m^2^. The median slope increases from 0.29 to 0.52 at elevations between 23 m and 48 m, it is approximately constant at 0.48 ± 0.01 at intermediate elevations between 55 m and 72 m and decreases to 0.32 again for higher elevations. The median hilltop curvature increases from 0.08 to 0.18 at 41 m and then decreases gradually. Median hillslope length has its highest value of 93 m at an elevation of 25 m and is approximately constant at 64.72 ± 4.69 m for higher values of elevation. Four sets of river terraces can be found in the area. These are at elevations of 25, 35, 50 and 80 m corresponding to ages of 1, 1.3, 2, and 6 ky, respectively^21^ (Fig. S4). The largest terrace in the region, named KT_1_, was formed as part of the third group and has been associated with ages 2kyBP.

The ratio of badland area positively correlates with surface velocity for surface velocities above 7 mm yr^− 1^ and reaches a maximum value of ~ 0.68 at a surface velocity of 39 mm yr^− 1^ (Fig. 2a). For median slope gradients above 27^o^, the ratio of badland area is also positively correlated with the median gradient, and reaches a maximum value of 0.41 at gradients of around 54^o^ (Fig. 2b), and decreases for higher gradients. The ratio of badland area increases from 24 m of elevation to a peak ratio of 0.13 at 40 m and decreases to 0.09 for elevations exceeding 60 m (Fig. 2c).

### Tectonic uplift controls the distribution of Badlands

**Fig. 3:**
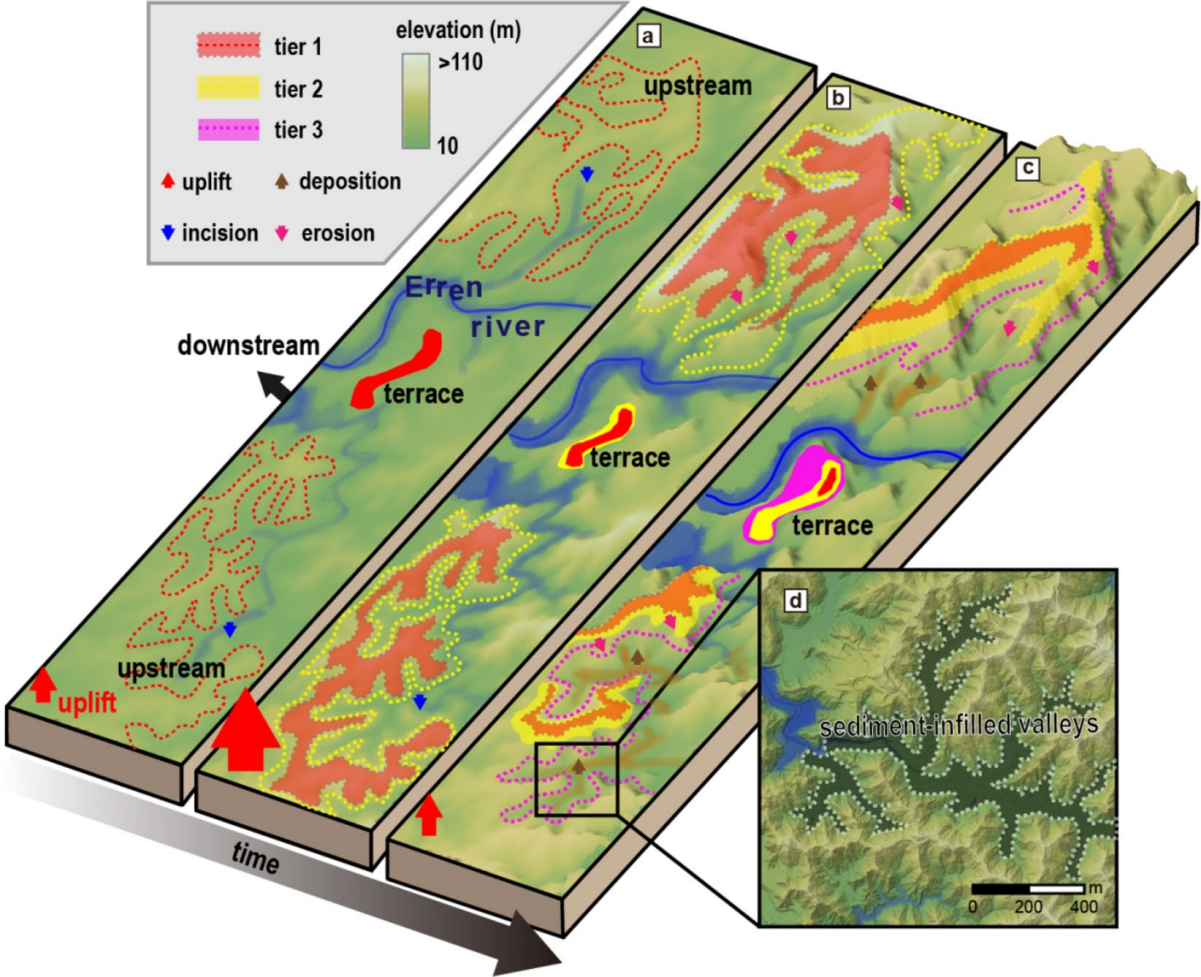
Schematic diagram of the development of mudstone badlands in response to uplift. The dashed line indicates the elevation of the badlands formed with the river terrace; the chronological sequence is distinguished by color. (**a**) Incision of the main stream (blue arrows) causes terrace formation. Lithologic weakness allows rapid upstream propagation of the incision signal. A first tier of steep slopes forms. (**b**) Rapidly increasing uplift causes tributary incision, which creates a second tier of steep barren slopes in the headwater regions (pink arrows). (**c**) Hillslope erosion prevails over decreasing uplift, thus forming sediment–infilled valleys (flat bottomed valley) on the toe of badlands (brown lines). (**d**) Color-shadow map of sediment–infilled valleys with 1 m spatial resolution.

The formation of badlands has been attributed to a high number of interacting variables, for example, the clay content of the regolith, earthquake–triggered landslides, deforestation, river migration, and land management practices^29–32^. We found that LOS surface velocity derived from SAR estimations positively correlates with the ratio of badland area (Fig. 2a), suggesting uplift related badland evolution. In addition, we found that badlands concentrate at elevations of ca. 40–60 m, corresponding to an age of 1.5–3 kyBP. This may imply that badlands developed during the same rapid uplift period that led to the formation of the KT_1_ terrace, the largest river terrace at the study site (cf. Figure 2c). Our interpretation can be summarized in a three–phase conceptual model. The first phase corresponds to the onset of uplift and its initial increase. The main stream incises and forms the first set of terraces. Tributaries respond by quickly incising, forming steep slopes along their channels (Fig. 3a). The second phase corresponds to the rapid increase of uplift until the maximum rate is reached. A second set of terraces forms around the time when the uplift rate is at its maximum. A second tier of steep slopes forms at an elevation below the first tier (Fig. 3b). Both tiers of steep slopes subsequently have developed into badlands by surface erosion and gully incision driven by heavy rainfall. In the third phase, the uplift rate decreases. Still, a third tier of steep slopes forms due to ongoing tributary incision (Fig. 3c). In contrast to previous phases, eroded hillslope material cannot be fully evacuated. It deposits in the upstream reaches of tributaries and turns them into sediment–infilled valleys (flat bottomed valley) (Fig. 3d). As a consequence of this conceptual model, badlands formed during the same phase have the same age and are located at the same height. Therefore, the topographic features of badlands, such as steep slopes and sharp ridges, should coexist with river terraces at the same elevation. We suggest that these three steps – corresponding to the onset of uplift, its maximum, its decline- and the associated geomorphic evolution provide a general sequence for badland formation in response to rapid tectonic uplift.

### Badlands as transitional landscapes controlled by uplift and erosion

**Fig. 4:**
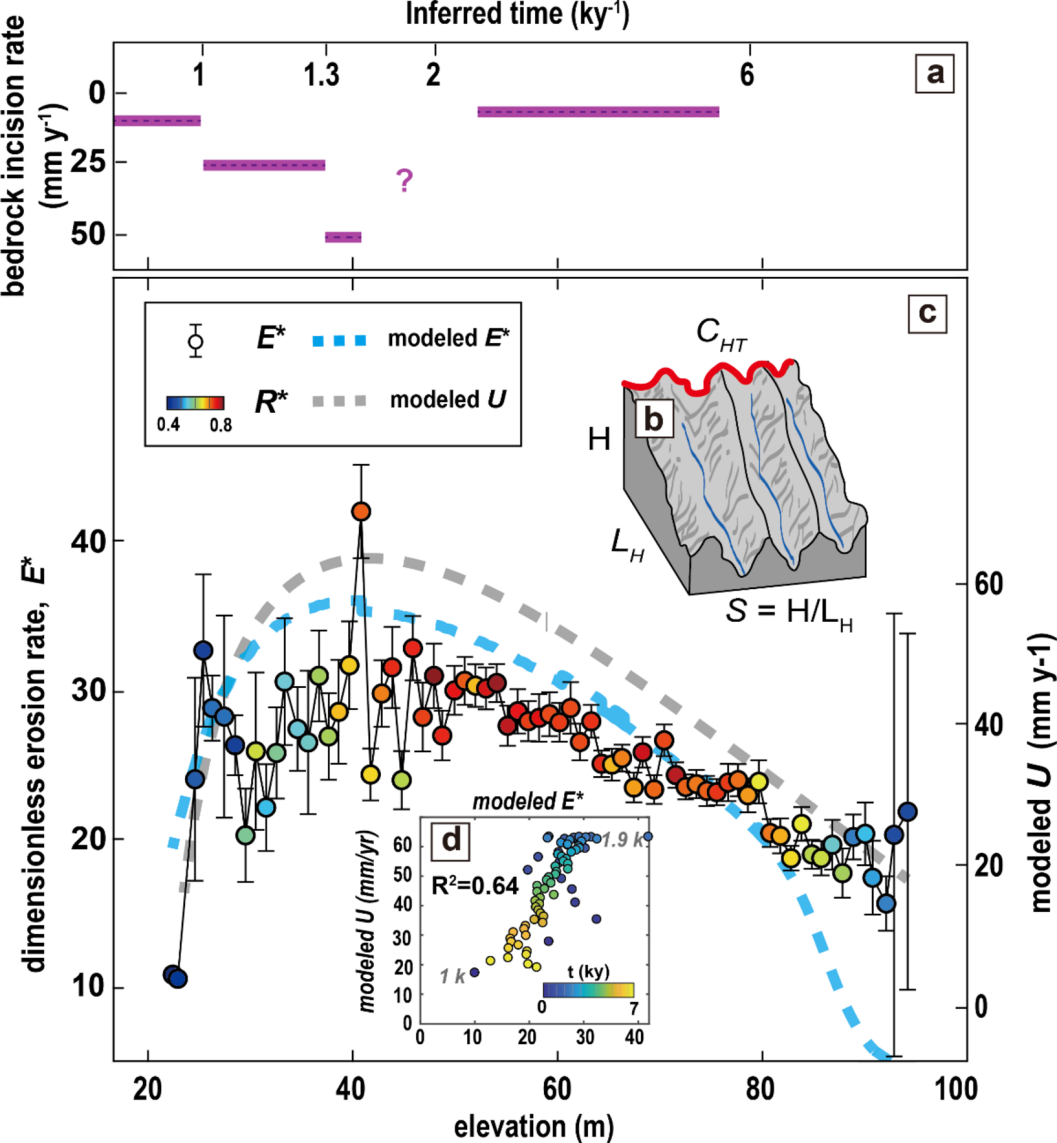
Inferential evolution of hillslope morphology. (**a**) Bedrock incision rate over the 6 kyBP, the elevation and the inferred time are matched based on C terrace dating^21^. (**b**) Sketch of parameterizations of badland hillslopes. (**c**) Variation in observed dimensionless erosion rate, E* (Eq. 3, obtained from elevation with respect to dated terraces) with elevation, with colours indicating the binned medians of dimensionless relief R* (Eq. 2). Error bars show the standard error of the mean for binned medians, and solid lines depict binned medians. The simple landscape evolution model (methods) yields modeled *U* (dashed grey line), calculated from Eq. (3), and modeled E* (dashed blue line). (**d**) Correlation of modeled E* and modeled *U*. Modeled *U* and modeled E* increase between 1 and 1.8 kyBP. Before 1.8 kyBP, uplift decreased, to which erosion showed a delayed response.

Hillslope erosion rates have been described by a nonlinear diffusion model^11,12^, controlled by hilltop curvature (*C*_*HT*_), slope length (*L*_*H*_) and mean hillslope gradient (*S*). Here, we incorporated the uplift timeline inferred from terrace ages into the nonlinear diffusion model to obtain modeled erosion, E* and modeled uplift rate, *U* (Fig. 4).

Observed E* from badland morphology, and modeled E* increased until ca. 1.5 kyBP (41 m of elevation), when the bedrock incision rate was highest, followed by a decrease (Fig. 4c). Similarly, the modeled *U* driven by observed E* increases from 1.7 to 6.2 cm y^− 1^ between 1 and 1.9 kyBP, followed by a decrease (Fig. 4d). The modeled *U* is comparable to the observed uplift rate, supporting the feasibility of reconstructing uplift rates from topography. Within the landscape archive framework^10–12^, E* in badland hillslopes can reflect the bedrock incision rate, indicating that badland hillslope erosion is linked to river incision. In line with our conceptual model, we suggest that badland formation is driven by tributary incision in response to uplift, which is reflected in its hillslope form and erosion rate.

In addition, dimensionless relief, R* is influenced by the high slope gradient (Fig. S4) and remains at high values above 0.7 between ca. 2 to 6 kyBP rather than before 2 kyBP (Fig. 4c), suggesting that the slope gradient might be influenced by erosion. Hilltop curvature, *C*_*HT*_ (Fig. S4) aligns with uplift history (Fig. 4c), indicating that it can effectively record uplift related topography^33^. The model predicts that hilltop curvature lags behind relief in its response to changing erosion rates, which makes it possible to distinguish growing from decaying landscapes. Given the correspondence of modeled *U* and modeled E* (Fig. 4d), we expect that fast–eroding badlands shorten the lag time of topographic response. In addition, they record the rate and timing of topographic response to perturbations during landscape transience.

## Conclusions

Present day surface velocity and hillslope gradient are positively correlated with the ratio of badland area in southwestern Taiwan (Fig. 2a-b), demonstrating the tectonically driven erosion could be expressed in hillslope morphology, mirroring the landscape characteristics in an active orogenic belt^34^. Elevations with high badland density and modeled erosion rates coincide with the history of rapid uplift at 1.3 to 1.5 kyBP (Fig. 2c), implying that hillslopes have recorded this tectonic event. If badlands can serve as a marker of uplift history, then our observations not only suggest that the existing geomorphic framework applies to erodible badlands but also demonstrate that the integrated change in hillslope morphology with elevation, along with comparable chronological data, can be used to quantify uplift rates between geodetic and geological time scales. Badland landscapes are a microcosm of natural landscapes, observations and numerical simulations based on the rapid hillslope adjustments to tributary incisions caused by tectonic activity can serve as a reference for the landscape evolution of almost all fluvial-connected hillslopes on Earth’s surface. Consequently, badlands in this study site serve as an example to address longstanding questions related to the recurrence cycle of geohazards and the evolution of transient landscapes in other rapidly uplifting regions. For example, the concentration of badlands at a particular elevation in the landscape may indicate the occurrence of an earthquake or a change in the activity of a fault.

## Methods

### Measurement of present surface uplift using the PSI method

To characterize present surface velocities (Fig. 1b), we collected 17 advanced land observing satellite synthetic aperture radar (ALOS SAR) images obtained by the Japan aerospace exploration agency (JAXA) from 2007 to 2010, and used the Stanford Method for Persistent Scatterers (StaMPS) software^35^ to compute line-of-sight (LOS) velocities based on the persistent scatterers interferometry (PSI) methodology. We used fine mode ascending images and excluded the image on March 12, 2010, which contains signals of coseismic and large postseismic displacements of the Jiashian earthquake on March 4, 2010 in southern Taiwan^36^. The average altitude of the satellite was 691.65 km and the angle between the satellite azimuth and the equator was 98.16°. We used single look complex (SLC) images and chose the image on January 20, 2008 as the master image. We followed the StaMPS procedure^35^ and selected a coherence value of 0.4 as the threshold to pick PS points. We analyzed a time series of 56 continuously–recorded GPS stations from the GPS lab at the Institute of Earth Science, Academia Sinica^37^, and calculated their secular velocities. We then projected the 3D GPS velocities onto the LOS direction and compared the projected GPS velocities to the average SAR velocities of all PS points with a 300 m radius around the GPS stations. Finally, we chose the GPS station AKND as a reference point and computed the average SAR velocities concerning the station.

### Extracting parameters from hillslope morphography

To reconstruct the long–term relationship between hillslope morphology and tectonic forcing, we used the landslide inventory database obtained from the Forestry and Nature Conservation Agency (https://www.forest.gov.tw/) based on satellite imagery for segment–based interpretation to delineate the extent of barren land. We extracted the slopes for drainage areas above 2000 m^2^, and the mean slope gradient above 5^o^ and manually filtered for artificial structures, i.e., farmland, buildings and roads (Fig. S1). We set the target zone for elevations from 20 m to 100 m to investigate the correspondence between uplift history and hillslope morphology, broadly including all badlands that concentrate at elevations of 50–80 m (Fig. S2 and Table S1). The ratio of badlands was defined as the badlands area divided by the area at the same contour, with values ranging from 0 to 1. For example, we binned the ratio of badlands using bin boundaries at every 1 mm y^− 1^ of LOS displacement and calculated the ratio of badland area within the binned area (Fig. 2a).

Roering et al. (2007)^28^ suggested a framework of dimensionless variables for analyzing hillslope morphology, consisting of dimensionless relief, R^*^, dimensionless erosion rate, E^*^, and dimensionless uplift. The dimensionless relief, R^*^, is defined by:

2 $$\:{R}^{*}=\frac{S}{{S}_{c}}$$

Here, *S* is the slope gradient in tangent value, *S*_*C*_ = 0.6 is a critical slope value. While this value is below *Sc* = 1.2 of Roering et al. (2007)^28^, the temporal evolution of E^*^ is more crucial for this study. Therefore, in the absence of better constraints, we choose 0.6 to allow E* to closely approximate the uplift rate. Dimensionless erosion rate, E^*^, is defined by:

3 $$\:{E}^{*}=\frac{2{C}_{HT}{L}_{H}}{{S}_{c}}$$

Here, *C*_*HT*_ is the curvature of the hilltop and *L*_*H*_ is the hillslope length.

We binned elevation data using bin boundaries at every 1 m of elevation and calculated the median of R* and E* for each bin (Fig. S4) The equations include three topographic features: (i) slope length (*L*_*H*_), is defined as the maximum horizontal distance from the outlet of the slope to the edge of the slope (Figure S3), (ii) slope (*S*), the ratio of a hillslope’s height to its length, and (iii) hilltop curvature (*C*_*HT*_), the profile curvature of the hillslope divide. All of these values were calculated for each extracted slope by using 1 m topographic data obtained from airborne LiDAR.

### Landscape evolution model for constraining E* and *U*

We designed a simple 1D numerical model to explore the theoretical response of hillslopes to different uplift forcing and generate time series of E*. Our simulations use the nonlinear diffusion equation originally formulated by Roering (1999)^38^:

4 $$\:\frac{dz}{dt}=\frac{d{q}_{s}}{dx}$$

and

5 $$\:{q}_{s}\:=\:\frac{\left(K\:S\right)}{\left(1\:-\:{\left(\frac{S}{{S}_{c}}\right)}^{2}\right)}$$

Where z is the elevation (*L*), t the time (*T*), qs the sediment flux (*L2/T*), *x* is the distance (*L*), *K* is the diffusivity coefficient (*L2/T*), S is the local gradient (*L/L*) and *Sc* the critical slope (*L/L*). This scheme predicts that the long–term evolution of a hillslope tends toward a critical slope *Sc* with a nonlinear increase of sediment flux as the slope tends toward *Sc*. This equation has been widely used in various forms in numerical simulations^39,40^.

The simple nature of our numerical experiments allows us to use a straight–forward centered finite difference scheme to solve the equation explicitly. We use a formulation adapted to run in 1D, as described by Eq. 6 of Perron (2011)^39^:

6 $$\:\frac{\left({z}_{x}^{t+1}-{z}_{x}^{t}\right)}{\varDelta\:t}=\:U\:+\:K\left[\frac{{z}_{x}^{t}}{\left(1\:-\:{\left(\frac{{z}_{x}^{t}}{{S}_{c}}\right)}^{2}\right)}\:+\:2\:\frac{\left({z}_{x}^{t2}{z}_{xx}^{t}\right)}{{S}_{c}^{2}\left(1\:-\:\frac{\left({Z}_{x}^{{t}^{2}}\right)}{{S}_{c}^{2}}\right)}\right]$$

where *U* is the uplift rate. To set up multiple uplift scenarios, we created an uplift history by linearly interpolating results based on the C dating data. The curve increases from the minimum uplift rate and approaches the maximum uplift rate at 8600 years, then follows the decrease until 10,000 years. After 10,000 years, we set the uplift rate as the minimum uplift rate until the end of the simulation. Note that the maximum uplift rate is set as 10 times the minimum uplift rate, for example, uplift rate = 28 mm/year means the scenario with 28 mm/year of maximum uplift rate and 2.8 mm/year of minimum uplift rate. Therefore, we can keep this time distribution of uplift and scale proportionally to different maximum uplift rates. In total, we conducted 25 simulations with maximum uplift rates ranging from 1 mm/year to 28 mm/year (Fig. S5a). Equation (3) is implemented in Python, using Kwan Lam et al. (2015)^41^ JIT compiler to optimize the speed of execution, as the explicit nature of the equation and the rather extreme uplift values tested force small timesteps to keep the scheme stable.

For the initial landscape, we run the model on a trapezoidal hillslope with a low uplift (< 0.1 mm y^− 1^) until it reaches a steady state, then we use the low–relief (elevation < 5 m ) steady–state hillslope profile with 180 m wide (Fig. S5b) to run our tests obtained by running the model until it reaches stable topography under low–uplift conditions. As such, we can obtain the time series of uplift rates and the corresponding modeled E^*^ (Fig. S5c). Note that a high diffusivity value of 0.5 was chosen to fit field observation. Our approach remains deliberately simple and intends to explore the theoretical trends in nonlinear hillslopes response to wide ranges of uplifts, as well as time series of modeled E*, rather than reproducing the exact topography observed on the field. Based on the 1D numerical models, the maximum modeled E^*^s had a statistically significant correlation with maximum uplift rates (Fig. S5d), which we can use to scale modeled E* to uplift rate.

To invert the uplift rate from hillslope morphology, we first idealized the observed E^*^ curve (Fig. 4c) as a Gamma function and determined the shape and scale factors of 1.2 and 98.4, respectively, using a Monte Carlo method. By using the Monte Carlo method, we can flexibly apply the method to any uncertain E* curve. To keep the shape of the idealized E* curve and make the maximum E* = 1 for converting to the maximum uplift rate, we normalized the idealized E* curve with values ​​between 0 and 1. Therefore, we can scale the normalized curve to the modeled uplift rate by the following conversion Eq. (7):

7 $$\:modeled\:uplift\:rate={Curve}_{normalized}\times\:(1.392\times\:{E}_{max}^{*}+3.397)$$

## Electronic supplementary material

Below is the link to the electronic supplementary material.

Supplementary Material 1

## Data Availability

Script for the landscape evolution model that supports the findings of this study has been deposited in Figshare https://doi.org/10.6084/m9.figshare.28355798. Other raw datasets are available from the corresponding author on reasonable request.
