# Supplementary material for: Badland distribution as a marker of rapid tectonic activity
Source: Sci Rep. 2025 May 7;15:15912. doi: 10.1038/s41598-025-00795-9 (PMC12059066; doi:10.1038/s41598-025-00795-9)
Supplement: Supplementary file 1 — Supplementary Material 1 [file 41598_2025_795_MOESM1_ESM.docx]

Supplementary Information for

**Badland distribution as a marker of rapid tectonic activity**

Contents of this File

Supplementary Figures. S1–7 and Table. S1.

**Data Sources of Airborne LiDAR DEM**

The airborne LiDAR DEM data were provided by the Satellite Survey Center, Department of Land Administration, Ministry of the Interior, and Central Geological Survey, Ministry of Economic Affairs. With the assistance of GPS, and IMU (Inertial Measurement Unit), each reach LiDAR return has positioning data. LiDAR data are composed of dense point clouds. The higher point cloud density can be used to build a high-density DEM. The LiDAR DEM used in this study was acquired in 2010. The grid spacing size is 1 m. Overlapping between swaths was greater than 40%. The point density of the areas where the elevation below 800 m is higher than 2 points/m^2^, whereas the elevation above 800 m is higher than 1.5 points/m^2^. The TWD97 projection was used. The external error evaluation of airborne LiDAR is to select a certain proportion of ground control points and LiDAR data for comparative analysis to evaluate the overall accuracy. Ground control areas were selected from bare land, dwarf vegetation (short grass, dwarf trees, tea gardens, etc.), sheltered forests, densely covered forests, metropolitan areas, etc. Each category selects about 30 points for the precise measurement of RTK-GPS or total station. The elevation difference between the ground point and LiDAR DEM is mostly within 10 cm (Hsieh et. al., 2017).

Reference

Hsieh, Y.-C.; Tang, C.-L.; Chiu, C.-L.; Chen, H.-J.; Fei, L.-Y.; Chen, M.-M.; Hou, C.-S.; Lin, C.-W.; Hu, J.-C., 2017, Potential Catastrophic Landslides Mapping and Surface Displacement Monitoring in The Disaster Areas Caused by Typhoon Morakot. J. Eng. Environ. 2017, 37, 18–44.

**The uplift mechanism of the Longchuan Fault**

Multiple observations along the Longchuan fault and the Chegualin fault indicate that this area has higher tectonic activity than plate tectonics alone. First of all, the higher tiers of Holocene river terraces in the anticline suggest regional uplift, which may be related to the development of underlying blind faults (Hsieh et al., 2001, 2002). Then, geological surveys have revealed that artificial structures near the Longchuan fault, such as bridges and roads, have been damaged due to fault deformation (Liu, 2013). Next, present-day geodetic data report a rapid tectonic uplift along the Longchuan fault and the Chegualin fault. GPS observations indicate that the surface velocity in this area exceeds the compression rate between the Eurasian Plate and the Philippine Sea Plate (Chang-Lee, 2014). Ching et al. (2016) utilized GPS velocity to invert the geometry and slip rate of the Hsiaokangshan fault and the Qishan fault, finding that the dislocation model results are less than the uplift rates on the hanging wall of the Longchuan fault. Thus, Ching et al. (2016) suggest that mud diapir could be an additional contributor to the deformation. Leveling surveys across the Longchuan fault indicate that the uplift rate decreases from 20 mm y^-1^ on the hanging wall to ca. 0 mm y^-1^ on the footwall (Huang et al., 2016). InSAR observations reveal a higher uplift on the hanging wall of the Longchuan fault (Chao, 2016; Pathier et al., 2014, Kuo, 2017). In addition, geodetic data and finite element numerical simulations conclude that the deformation mechanism is controlled by the duplex structure in the footwall of the Chegualin fault and a west-dipping fault on a depth of 4 km, with the high-pressure layers of the Gutingkeng mudstone (Kuo, 2017).

In summary, the mechanism of this tectonic uplift is still under discussion. Currently, at least, three explanations have been proposed: mud diapir (e.g., Ching et al., 2016), fault deformation (Hsieh et al., 2001, 2002), and the duplex structure in the footwall of the Chegualin fault combined with the distribution of high–pressure layers in the Gutingkeng mudstone (Kao, 2017). However, river terraces and geodetic data confirm the region and rate of rapid tectonic uplift in the study area. Given that the uplift pattern has been reported, we suggest that the dataset used in this study is sufficient to support the main focus of our study—exploring how tectonic uplift is recorded in the badland landscape.

Reference

1. Chang–Lee, C. Behavior analysis of the LCNF–CHNF fault system using geodetic measurements. Master thesis, Department of Geomatics, National Cheng Kung University. (2014) (in Chinese).
2. [Chao](https://ndltd.ncl.edu.tw/cgi-bin/gs32/gsweb.cgi/ccd=crZxCO/search?q=aue=%22Chuan-MinChao%22.&searchmode=basic), C.–M. Surface Creeping Analysis of the Fengshan Fault in SW Taiwan from GPS observations and PSInSAR. Master thesis, Department of Geomatics, National Cheng Kung University. (2016). (in Chinese).
3. Ching, K. –E., Gourley, J. R., Lee, Y. –H., Hsu, S.-C., Chen, K. –H., Chen, C.-L. Rapid deformation rates due to development of diapiric anticline in southwestern Taiwan from geodetic observations. *Tectonophysics* **692**, 241–251 (2016).
4. Hsieh, M. –L., Knuepfer, P. L. K. Middle–late Holocene river terraces in the Erhjen River basin, southwestern Taiwan-implications of river response to climate change and active tectonic uplift. *Geomorphology* **38**, 337–372 (2001).
5. Hsieh, M. –L., Knuepfer, P. L. K. Synchroneity and morphology of Holocene river terraces in the southern Western Foothills, Taiwan: A guide to interpreting and correlating erosional river terraces across growing anticlines. *Geol.Soc. Am. Bull.*, 55–74. (2002).
6. Huang, C., Kao, Y. –P., Tung, H., Huang, H. –W., Hu, J. –C., Huang, M.-H. Co-Seismic Deformation of the 2016 Meinong Earthquake, and the Interseismic Crustal Deformation of Southwestern Taiwan. *Geotech*. **148**, 25–30. (2016). (in Chinese).<https://tpl.ncl.edu.tw/NclService/JournalContentDetail?SysId=A16021564>
7. Kao, Y. –P. Anomalous high deformation rate in mudstone of fold-and-thrust belt in SW Taiwan. Master thesis, Department of Geosciences, National Taiwan University. (2017). (in Chinese).
8. Liu, Y. –C. Geological survey of Longchuan fault. 2012 Annual Performance Presentation of the Central Geological Survey, Ministry of Economic Affairs, **32** (2013). (in Chinese).
   chrome-extension://efaidnbmnnnibpcajpcglclefindmkaj/https://twgeoref.gsmma.gov.tw/GipOpenWeb/imgAction?f=/2013/20131533/0032.pdf
9. Pathier, E., Fruneau, B., Doin, M. P., Liao, Y. T., Hu, J. –C. Champenois, J. What are the tectonic structures accommodating the present-day tectonic deformation in South-Western Taiwan? A new interpretation from ALOS-1 InSAR and GPS interseismic measurements. Geodynamics and Environment in East–Asia. 7^th^ France–Taiwan Earth Sciences Symposium, Hualien, Taiwan. (2014).

**Extraction of drainage basins**

We used TopoToolbox, a Matlab-based tool (Schwanghart et al., 2014), to extract the boundaries of drainage basins.

**
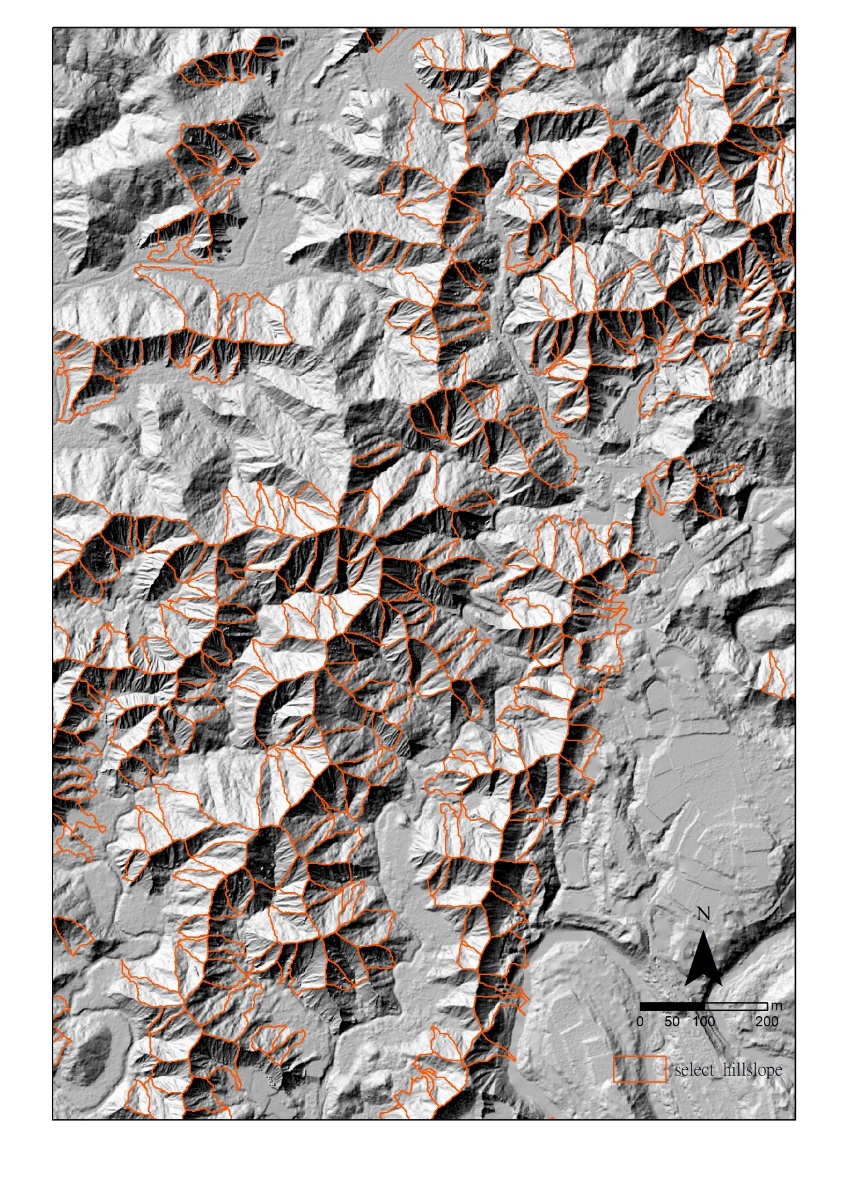
**

**Figure S1. The boundaries of drainage basins with 1 m spatial resolution hillshade map.** Orange areas denote boundaries of drainage basins which are defined as the slopes for drainage areas above 2000 m^2^ and mean slope gradient above 5^o^, and manually filtered for farmland, buildings, and roads.

Reference

Schwanghart, W., Scherler, D., 2014, Short Communication: TopoToolbox 2-MATLAB-based software for topographic analysis and modeling in Earth surface sciences. Earth Surf. Dyn. 2, 1–7.


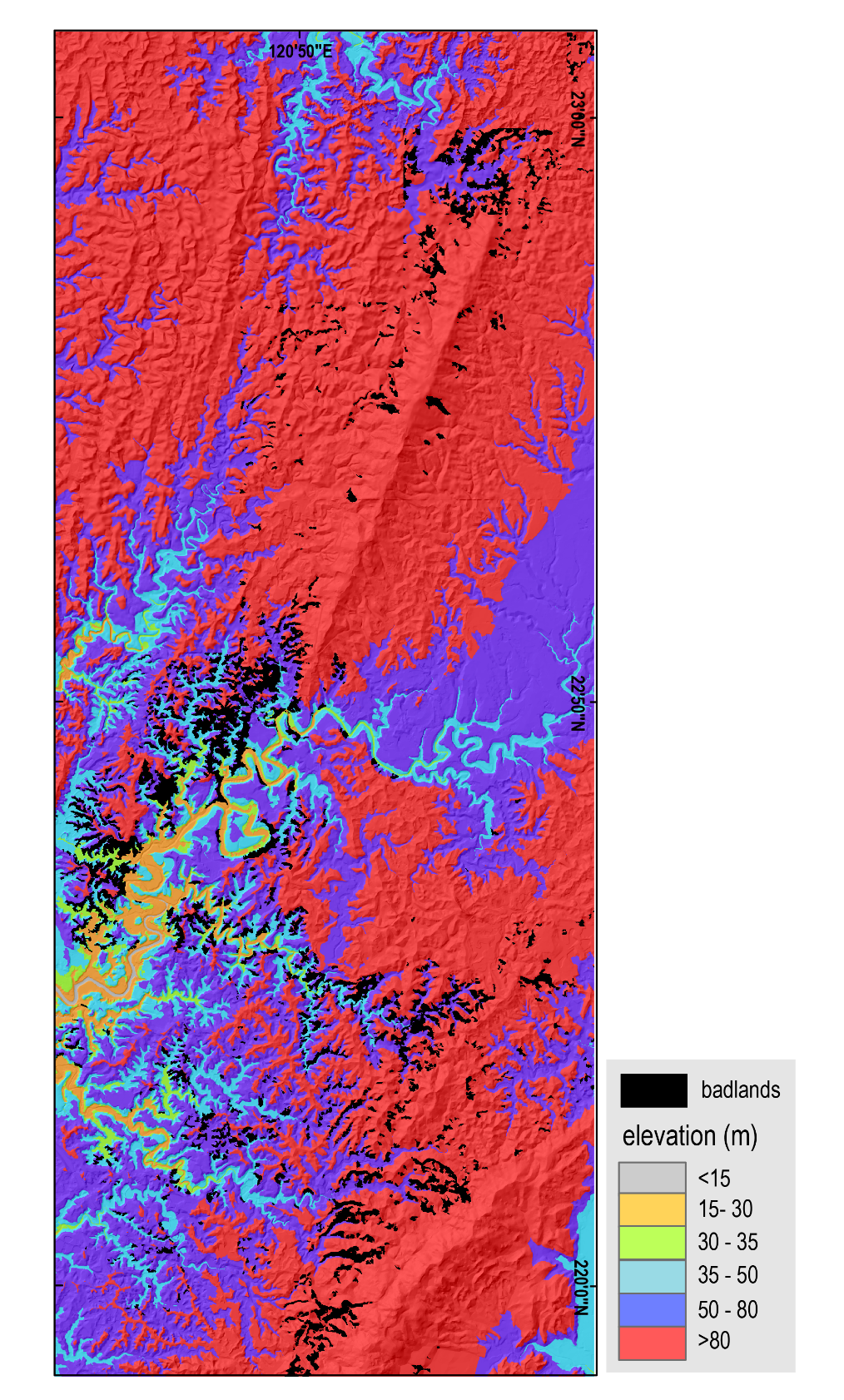


**Figure S2. Hypsometric map with hillshade of the study site and distribution of badlands.** The colors are classified according to the height of the river terrace published by Hsieh and Knuepfer (2001). Gray color is referred to be less than 15 m a.s.l.; Orange color is referred to 15–30 m a.s.l.; Green color is referred to 30–50 m a.s.l.; Sky blue color is referred to 35­–50 m a.s.l.; Indigo color is referred to 50-80 m a.s.l.; red color is referred to higher than 80 m a.s.l. The position of Hy, KT1 and KT2 terraces are located on elevations of 75, 50 and 35 m a.s.l., respectively.

Table S1. THE AREA OF BADLANDS ALONG ELEVATION

| Class | Total area  (km^2^) | Badland area  (km^2^) | Badland area ratio  (%) |
| --- | --- | --- | --- |
| <15 m | 0.07 | 0.00 | 0.00 |
| 15–30 m | 2.86 | 0.15 | 5.23 |
| 30­–35 m | 2.47 | 0.25 | 10.12 |
| 35­–50 m | 12.21 | 1.44 | 11.81 |
| 50-80 m | 44.96 | 4.53 | 10.08 |
| >80 m | 74.70 | 5.52 | 7.39 |


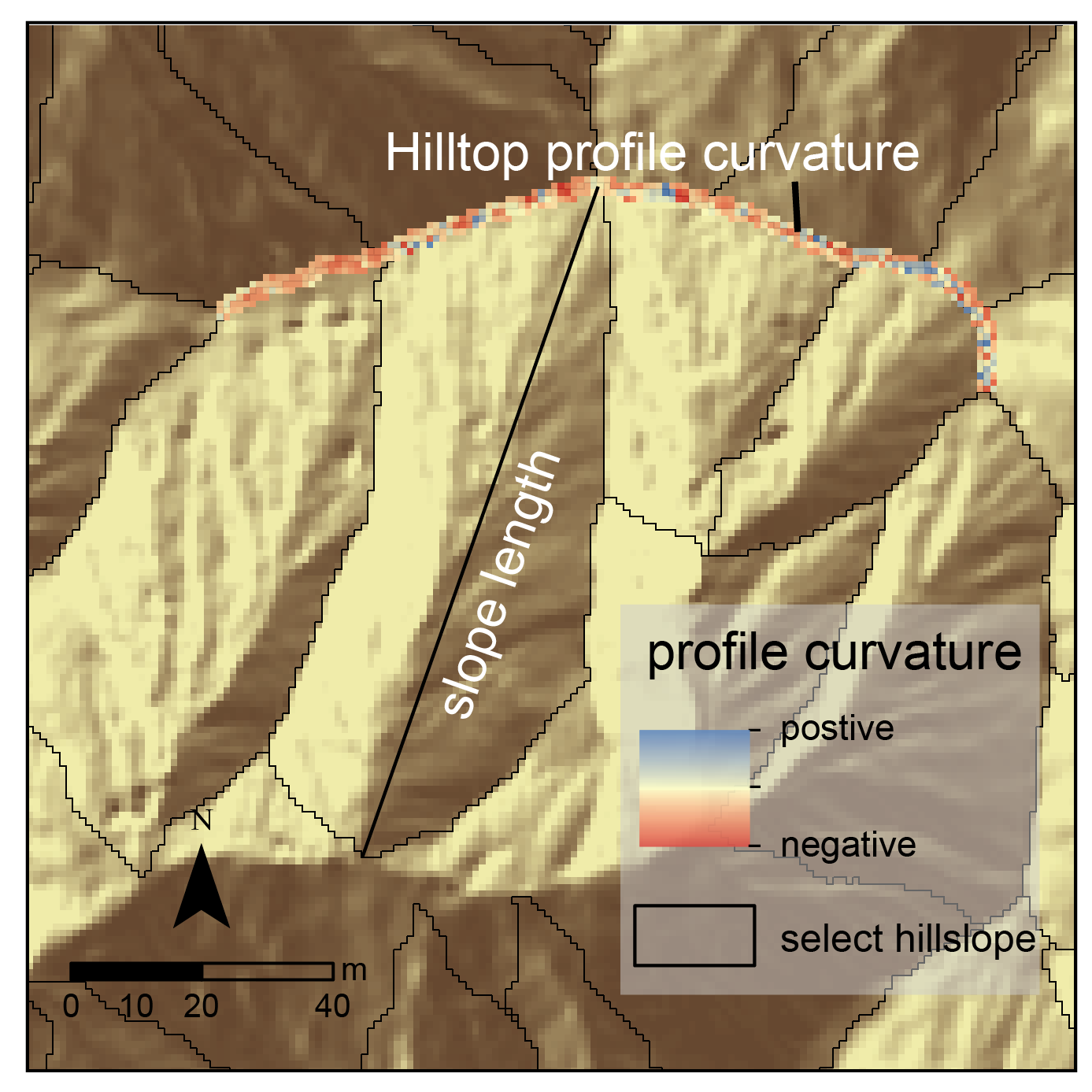


**Figure S3. Examples of extracting hilltop profile curvature and slope length. Hilltop curvature refers to the profile curvature of the hillslope divide.** Hilltops were extracted using the method of Xie et al. (2004). Slope length refers to the longest length of the projection hillslope, and the longest length is defined as the maximum value of the Euclidean distance from the drainage outlet of the hillslope.

Reference

Xie, M., Esaki, T., Zhou, G. 2004, GIS-Based Probabilistic Mapping of Landslide Hazard Using a Three-Dimensional Deterministic Model. Natural Hazards 33, 265–282. https://doi.org/10.1023/B:NHAZ.0000037036.01850.0d


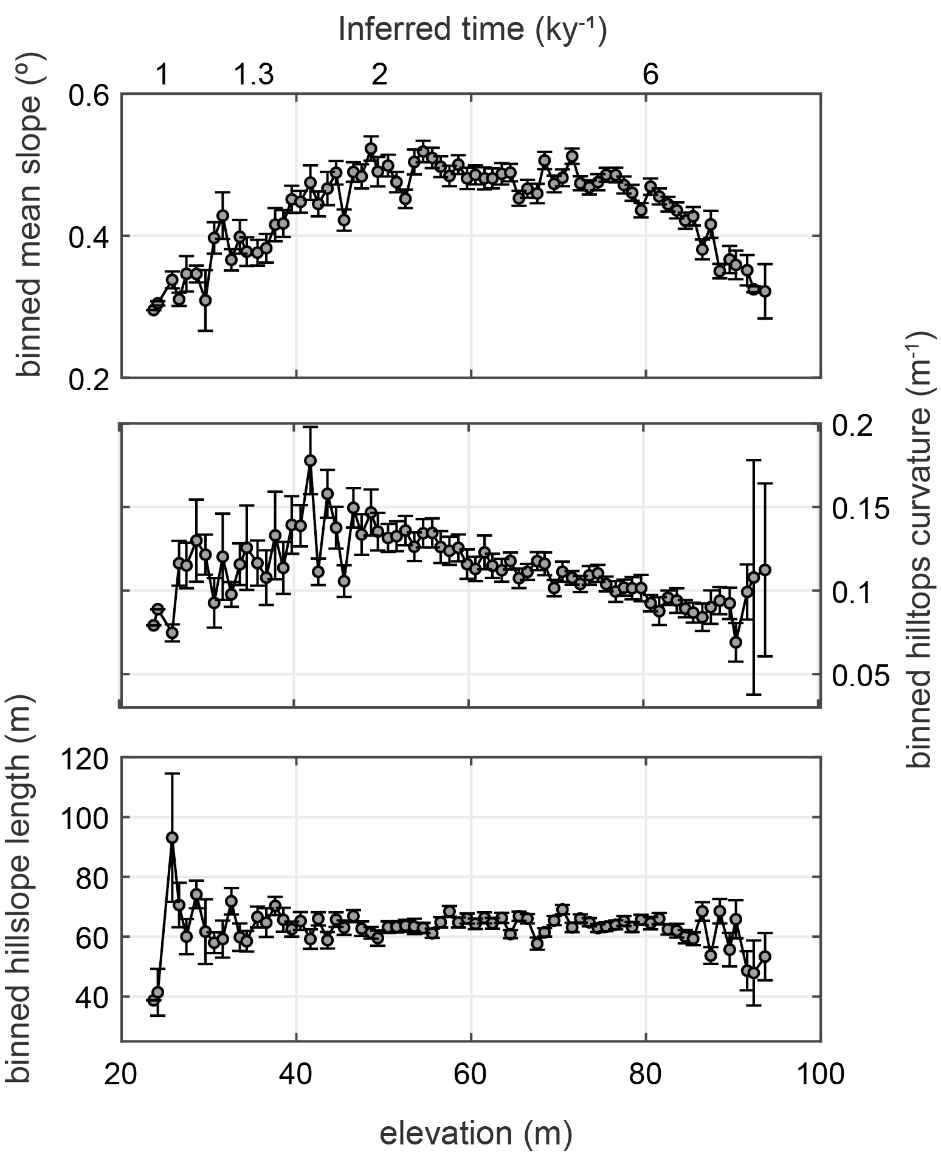


**Figure S4. Distribution of hillslope morphology.** Distribution of surface metrics sampled from hillslopes (Fig. 1). Error bars show standard errors of the mean for binned means, and black solid lines show the binned medians. The gradient is calculated by computing the steepest downward gradient of a digital elevation model using an 8 neighborhood cell. The space-for-time substitution is based on river bedrock incision rates (Hsieh and Knuepfer, 2001).

Reference

Hsieh, M.-L., and Knuepfer, P.L.K., 2001, Middle-late Holocene river terraces in the Erhjen River basin, southwestern Taiwan-implications of river response to climate change and active tectonic uplift. Geomorphology, 38, 337–372. https://doi.org/10.1016/S0169-555X(00)00105-7


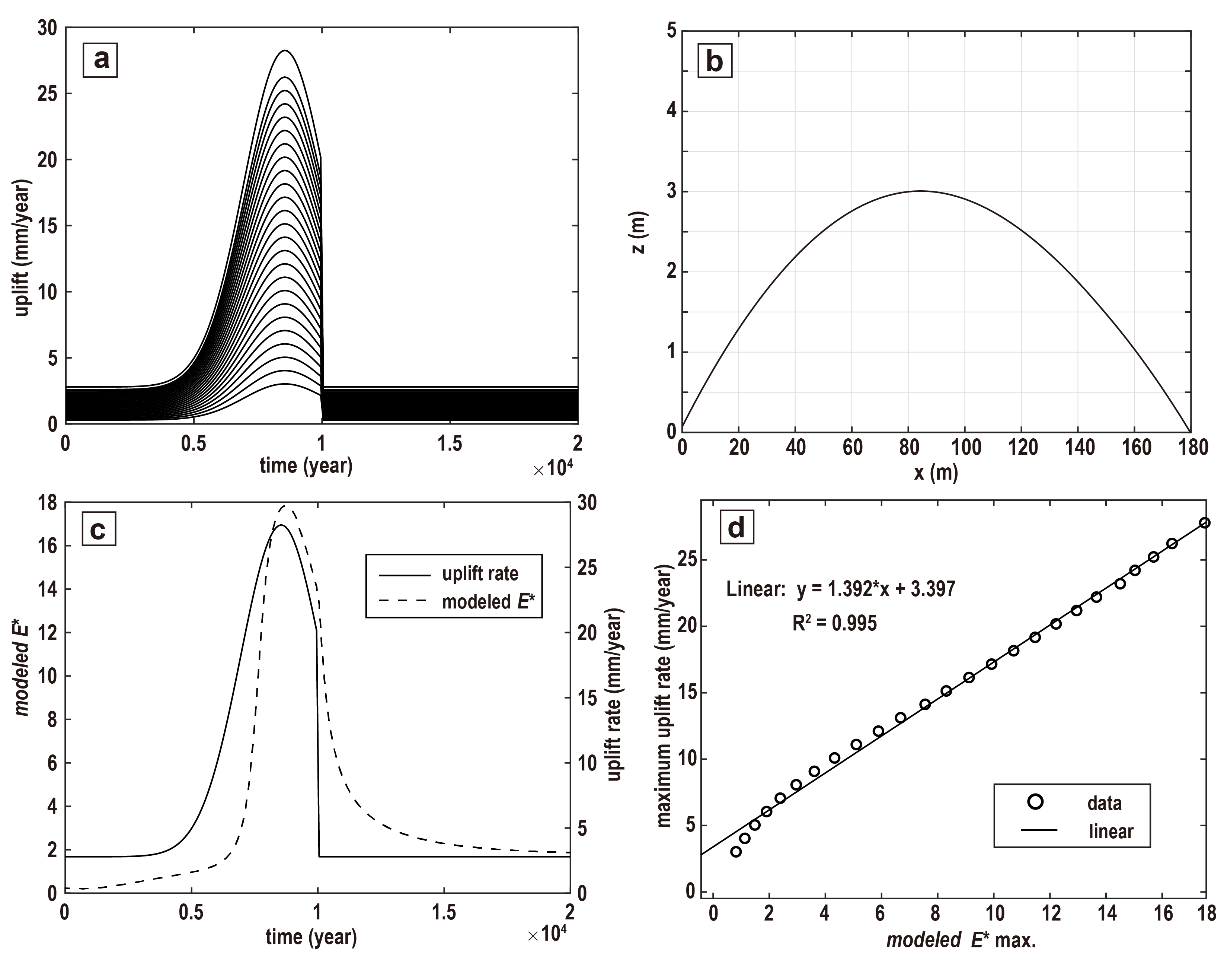


**Figure S5. 1D landscape evolution model.** (a) the setting of the time-series uplift rate. (b) the initial landscape of the 1D landscape evolution model. (c) the example of a time series of uplift rate and modeled *E** from maximum uplift rate = 28 mm/year. (d) correlation between maximum modeled *E** and maximum modeled uplift rate, black dots denote the values from 25 simulation results, and black line denotes the fitting curve.


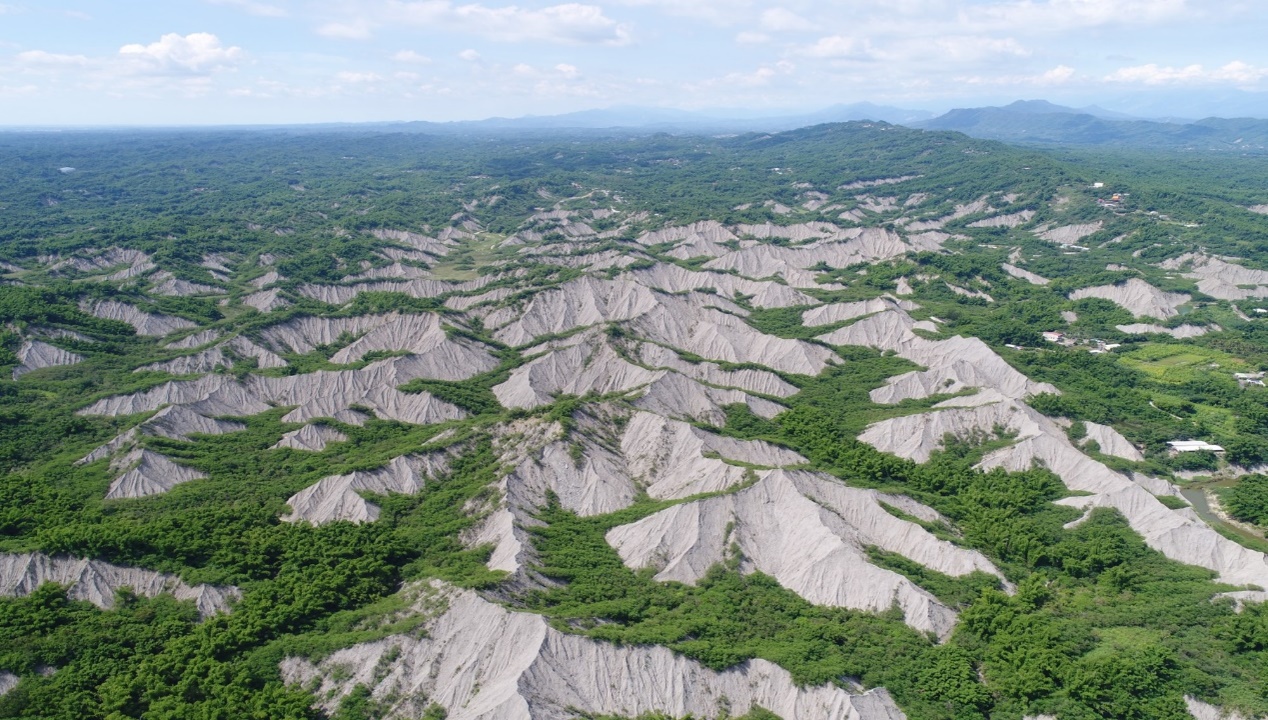


**Figure** **S6**. Aerial view of the mudstone badlands landscape, with a view toward Tienliao (Fig. 1a).

**Annual precipitation and lithological proportions in non-badland and badland areas of the study site**

The average annual rainfall in the non-badlands area is 2176±72 mm, and the average annual rainfall in the badlands area is 2182±38 mm (Figure S7(a)), within error of each other. In addition, soft rock, such as alluvial soil, mudstone, shale, laterite, and sandy shale are the main lithology of the study site (Figure S7(b)). Mudstone has the highest proportion, making up 61% and 78% of the badland and non-badland areas, respectively. Therefore, lithology cannot be the main contributing factor to badland distribution in our study site. Tectonic structures in this region trend in an NNE-SSW direction, with well-developed faults and folds in the same orientation. Vertical dipping strata can be observed on both sides of the faults, exposing weak mudstone and shale at the surface, which are further subjected to fluvial and rain erosion (Lee, 1976). Additionally, the slope aspect influences the hillslope-scale badlands distribution. For example, aerial photographs document that 74.6% of the 208 badland slopes in the same study area are south-facing (Chen et al. 1984). Soil temperatures on south-facing slopes are significantly higher than on north-facing vegetated slopes due to greater solar radiation (Hsu, 1987). Without vegetation protection, the increased heat absorption and disproportionate evapotranspiration not only create an unfavorable environment for vegetation growth but also lead to the extensive development of desiccation cracks (Yang et al., 2021).
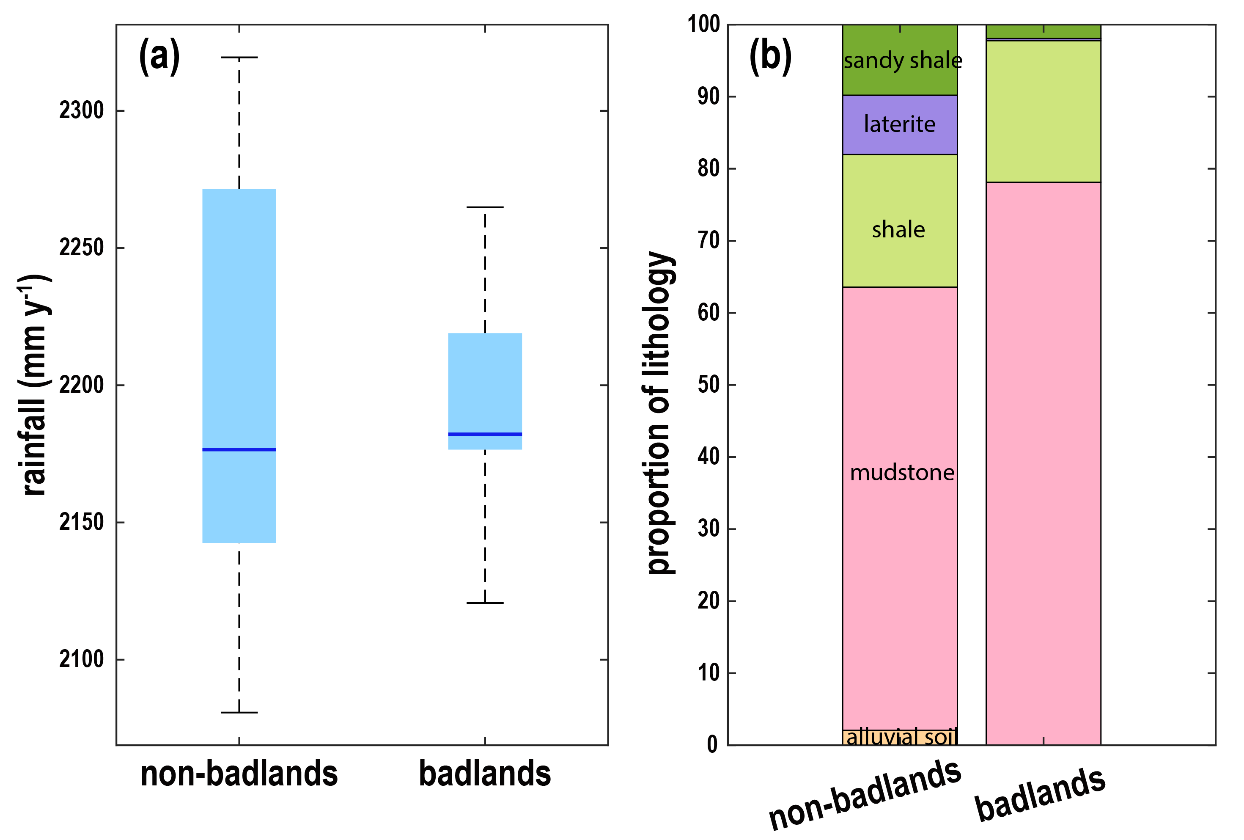


Figure S7. Annual precipitation and lithological proportions in non-badland and badland areas of the study site. (a) Annual precipitation in non-badland and badland areas. (b) lithological proportions in non-badland and badland areas.

Reference

1. Lee, M.-C. (1976) Geomorphological Research On The Typical Badlands In Southwestern Taiwan, Geographical Research(2), pp.201-224. (in Chinese).
2. Hsu, H.-H. (1986) An investigation of the micro-meteorological characteristics of the mudstone area in southwestern Taiwan, Phase I, Disaster Prevention Investigation. Natl. Sci. Counc., Taipei, Taiwan, R.O.C., Rep. 75-12. (in Chinese).
3. Yang, C.-J et al (2021) Quantification of mudcracks-driven erosion using terrestrial laser scanning in laboratory runoff experiment, Geomorphology (375) https://doi.org/10.1016/j.geomorph.2020.107527.
4. Chen, S.-T. (1984) A Study On The Erosion Characteristics Of The Mudstones In The Southwestern Taiwan (I). Natl. Sci. Counc., Taipei, Taiwan, R.O.C., Rep. 73-70. (in Chinese).
